# Noise reduction using a Bayesian penalized-likelihood reconstruction algorithm on a time-of-flight PET-CT scanner

**DOI:** 10.1186/s40658-019-0264-9

**Published:** 2019-12-10

**Authors:** Paulo R. R. V. Caribé, M. Koole, Yves D’Asseler, B. Van Den Broeck, S. Vandenberghe

**Affiliations:** 1 Medical Image and Signal Processing – MEDISIP, Ghent University, Corneel Heymanslaan 10, 9000 Gent, Belgium; 2 Division of Nuclear Medicine and Molecular Imaging, UZ/KU, Herestraat 49, B-3000 Leuven, Belgium; 3 Department of Nuclear Medicine, Ghent University Hospital, Corneel Heymanslaan 10, 9000 Gent, Belgium

**Keywords:** Penalized-likelihood reconstruction, BSREM, Q.Clear, OSEM, PET

## Abstract

**Purpose:**

Q.Clear is a block sequential regularized expectation maximization (BSREM) penalized-likelihood reconstruction algorithm for PET. It tries to improve image quality by controlling noise amplification during image reconstruction. In this study, the noise properties of this BSREM were compared to the ordered-subset expectation maximization (OSEM) algorithm for both phantom and patient data acquired on a state-of-the-art PET/CT.

**Methods:**

The NEMA IQ phantom and a whole-body patient study were acquired on a GE DMI 3-rings system in list mode and different datasets with varying noise levels were generated. Phantom data was evaluated using four different contrast ratios. These were reconstructed using BSREM with different *β*-factors of 300–3000 and with a clinical setting used for OSEM including point spread function (PSF) and time-of-flight (TOF) information. Contrast recovery (CR), background noise levels (coefficient of variation, COV), and contrast-to-noise ratio (CNR) were used to determine the performance in the phantom data. Findings based on the phantom data were compared with clinical data. For the patient study, the SUV ratio, metabolic active tumor volumes (MATVs), and the signal-to-noise ratio (SNR) were evaluated using the liver as the background region.

**Results:**

Based on the phantom data for the same count statistics, BSREM resulted in higher CR and CNR and lower COV than OSEM. The CR of OSEM matches to the CR of BSREM with *β* = 750 at high count statistics for 8:1. A similar trend was observed for the ratios 6:1 and 4:1. A dependence on sphere size, counting statistics, and contrast ratio was confirmed by the CNR of the ratio 2:1. BSREM with *β* = 750 for 2.5 and 1.0 min acquisition has comparable COV to the 10 and 5.0 min acquisitions using OSEM. This resulted in a noise reduction by a factor of 2–4 when using BSREM instead of OSEM. For the patient data, a similar trend was observed, and SNR was reduced by at least a factor of 2 while preserving contrast.

**Conclusion:**

The BSREM reconstruction algorithm allowed a noise reduction without a loss of contrast by a factor of 2–4 compared to OSEM reconstructions for all data evaluated. This reduction can be used to lower the injected dose or shorten the acquisition time.

## Background

Fluorodeoxyglucose (FDG) PET/CT scans provide 3D images of metabolic activity combined with the anatomic structure. This functional imaging modality is widely used for cancer diagnosis in the initial stage and to determine the severity or to assess treatment response [1, 2]. PET/CT technology is constantly being improved and new systems are also combined with emerging improvements in image reconstruction. This leads to changes in the resulting images which need to be tested and clinically validated. The resolution, noise, and quantitative accuracy of PET are not only affected by the hardware but also highly influenced by the reconstruction method. Nowadays, the most commonly used PET image reconstruction algorithm in clinical practice is a statistical iterative method known as the maximum likelihood expectation maximization (MLEM) [3–5]. This is a slowly converging method, but images are obtained in clinically acceptable times with acceleration through the use of subsets in ordered subsets expectation maximization (OSEM). However, this accelerated convergence can be problematic since the best result tends to oscillate between different subsets. One of the advantages of statistical reconstruction techniques is the ability to better model the emission and detection process [6]. The effects of attenuation, detector normalization, and contamination by scattering and randoms are nowadays corrected in the reconstruction algorithm. These improved models of the interaction in patient and system lead to a more quantitative final image. In the latest systems, the modeling of point spread functions (PSF) and time-of-flight (TOF) information have also been included and this has shown to lead to a major improvement in image quality [6, 7]. However, OSEM is also suffering from noise increase with an increasing number of iterations. In order to reduce image noise, the OSEM algorithm is usually stopped before contrast convergence occurs, in order to prevent excessive image noise amplification. In clinical practice, the algorithm is stopped after 2–4 iterations and 20–30 subsets. Additionally, these images are typically post-smoothed after reconstruction using a low-pass filter to remove noise levels and Gibbs artifacts at edges because of resolution modeling [8–10]. A new Bayesian penalized likelihood reconstruction algorithm which uses a block sequential regularized expectation maximization as an optimizer was introduced in the last few years by GE Healthcare. The algorithm, named Q.Clear on their PET scanners, is introduced to improve clinical image quality. The algorithm is expected to reach convergence without increasing noise while preserving edges [11]. Thus, instead of the kernel filter, image characteristics are determined by a regularization *β*-parameter which penalizes relative differences between neighboring pixels avoiding excessive smoothing over large edges. Also, Gibbs artifacts from resolution modeling are avoided [12]. Several research groups have investigated the improvements of the OSEM and BSREM reconstruction algorithms [13–17] but not with regards to image quality acquired on the new Discovery MI with 3-rings (GE Healthcare) silicon photomultiplier-based TOF-PET/CT with sensitivity of 7.3 cps/kBq and axial FOV of 15 cm. The lower sensitivity can be compensated for by using more activity or longer acquisition times; however, this is not always possible due to practical, financial, or dosimetric constraints. In this study, the performance and clinical use of BSREM was compared to OSEM with full modeling of PSF and TOF information for both algorithms acquired on the new Discovery MI with 3-rings (axial FOV of 15 cm). Both phantom and patient data were analyzed with regards to CR, background COV, CNR, SUV ratio, metabolic active tumor volumes (MATVs), and SNR. The aim of this study was to evaluate different *β*-factors compared to a clinical post-filter kernel for different datasets with varying noise levels to investigate whether and to what extent noise can be reduced by using BSREM instead of OSEM.

## Methods

### PET/CT system

All data were acquired on a digital GE Discovery MI PET/CT (DMI) system, installed in Ghent University Hospital, Belgium. The investigated system consists of three detector rings; each PET ring uses 136 detector blocks containing a 4 × 9 array of lutetium-based scintillator (LBS) crystals coupled to a 3 × 6 array of silicon photomultipliers (SiPMs) with Anger multiplexing for crystal identification [18]. Table 1 contains a summary of important design and performance parameters [19].

**Table 1:** Design and performance specifications of the GE Discovery MI commercial system

GE Discovery MI	Three detector rings
Axial FOV	15 cm
Patient bore size	70 cm
Photodetector	SiPM
Scintillator	LBS^a^ (LYSO)
Crystal element size	3.95 × 5.3 × 25 mm^3^
Coincidence timing window	4.9 ns
Sensitivity	7.5 cps/kBq
Spatial resolution (FWHM mm)	Radial/tangential/axial
@1 cm	4.69/4.08/4.68
@10 cm	5.58/4.64/5.83
@20 cm	7.53/5.08/5.47
Scatter fraction	41.7%
Peak NECR	102.3 kcps @ 24.7 kBq/ml
Clinical NECR	29.6 kcps @ 2.4 kBq/ml

^a^Lutetium-based scintillator

A well-countered cross-calibration scan was performed with ^18^F in a uniform cylindrical phantom before starting the tests as a common quality control and assurance procedure.

### Image reconstruction

The phantom and patient data were reconstructed using a matrix size of 256 × 256 with a slice thickness of 2.78 mm and multiple acquisition times for the two algorithms, respectively. BSREM (Q.Clear) reconstruction was done for different penalization *β*-factors 300, 400, 500, 600, 750, 1000, 1500, and 3000. These reconstructions were compared to OSEM reconstruction with three iterations, 16 subsets and a Gaussian post-filter with FWHM of 5.0 mm, as recommended by the manufacturer to be used in a clinical setting. All reconstructions included attenuation and scatter correction based on CT as well as PSF modeling and TOF information.

### Phantom data

The NEMA (National Electricals Manufacturers Associations) IEC image quality phantom was used for these experiments. To simulate lesions of different sizes, the phantom has six fillable spheres of different diameters (10 mm, 13 mm, 17 mm, 22 mm, 28 mm, and 37 mm). The phantom contains a lung insert, which consists of a cylinder positioned in the center of the phantom with an inner diameter of 44.5 mm and a volume of 194 ml. The lung insert was filled with low-density styrofoam pellets and pure water to simulate human lung tissue. The phantom was prepared according to the NEMA NU 2-2012 protocol [20]. The background volume of the phantom was filled with an activity concentration of 5.3 kBq/ml (52 MBq of ^18^F for 9800 ml). The sphere-to-background ratios were chosen to be 8:1, 6:1, 4:1, and 2:1 for the six spheres.

The phantom data experiments were obtained during a single bed position scan of 20 min in the full FOV of the TOF PET/CT. The central slice contained the six spheres and the adjacent slices were also used for the background ROIs. Sixty background ROIs of each slice thickness (12 ROIs on each of five slices) were drawn on the slices as close as possible to ± 1 cm and ± 2 cm on either side of the central slice. The CRs were determined for each hot sphere *j* by

$$ {\mathrm{CR}s}_{H,j}=\frac{\left({C}_{H,j}/{C}_{B,j}\right)-1}{\left({a}_H/{a}_B\right)-1},\kern14.25em (1) $$

where *C*_*H*, *j*_ is the average number of counts in the ROI in the transverse image slice that contains the center of the sphere *j*. *C*_*B*, *j*_ represents the average number of counts in the background ROI for sphere *j*. The terms *a*_*H*_ and *a*_*B*_ are the actual activity concentrations in the hot spheres and background respectively. The background COV was calculated as

$$ \mathrm{Backgroun}{\mathrm{d}}_{{\mathrm{COV}}_j}=\left(\frac{{\mathrm{SD}}_j}{C_{B,j}}\right)\kern14.5em (2) $$

where SD_*j*_ is the standard deviation and *C*_*B*, *j*_ is the average of all counts for of the 60 background ROI counts for sphere *j* [20]. Contrast-to-noise ratio was defined as CR divided by the background variability (calculated as described in [20]). The SUV values were obtained using a VOI drawn on the OSEM reconstruction of 20 min acquisition and then propagated to the BSREM reconstructions.

The CR data, CNR, and background COV of the phantom data were obtained in a single bed position with full 20 min acquisition. Datasets of 10, 5.0, 2.5, and 1.0 min, representing shorter scans with lower count statistics, were obtained using list-mode selections. Reconstructions of the phantom data were analyzed using the NEMA NU 2–2012 protocol and compared based on different reconstruction parameters.

### Clinical data

In our institution, an informed patient consent and a positive advice by the ethics committee are necessary for retrospective studies. The Belgian registration number (*Belgisch Registratienummer*) for this study is B670201939137. Images were analyzed according to FDG PET-CT European Association of Nuclear Medicine (EANM) procedure guidelines for tumor imaging [21]. The images were analyzed using OsiriX MD 10.0 tools fully optimized for a macOS Mojave 10.14 system installed in Ghent University Hospital, Belgium. A 71-year-old patient with multiple B cell lymphoma lesions of different sizes was selected for this study. The patient fasted at least 6 h before receiving an intravenous application of 340 MBq of ^18^F-FDG. Before the injection of the radioactivity tracer, a blood sample was taken to ensure the blood glucose levels (97 mg/dl). FDG-PET/CT imaging started 60 min after the intravenous injection of FDG. The total acquisition time (nine time lengths per bed-position) was 10 min (1.07 min/bp). From this dataset, scans of 5.0 min (0.34 min/bp) and 2.5 min (0.17 min/bp) were generated in list mode. The TOF-PSF-OSEM image with post-smoothing with a 5.0-mm filter was chosen as reference lesion volumes (VOIs). The VOIs were delineated using the 41% threshold of the maximum voxel value and then propagated to the BSREM reconstructions. The same reconstruction parameter settings were used as for the phantom data. The noise level was calculated as standard deviation (SD) divided by the SUV_mean_ of a large spherical reference volume (⌀, 3.0 cm) placed in the liver (normal uptake). The lesion SNR was computed as the difference between the SUV_mean_ of the lesion VOI and the background SUV of the reference VOI placed in the liver, divided by the SD of the value in the reference VOI. Contrast was calculated as lesion SUV_mean_ divided by SUV_mean_ of the liver reference VOI. The signal to noise was evaluated for the different lesions by comparing it to the noise level in the liver. The MATVs was evaluated as the lesion SUV_mean_ multiplied by the volume of the lesion.

## Results

### Phantom data

#### Contrast recovery

**Fig. 1:**
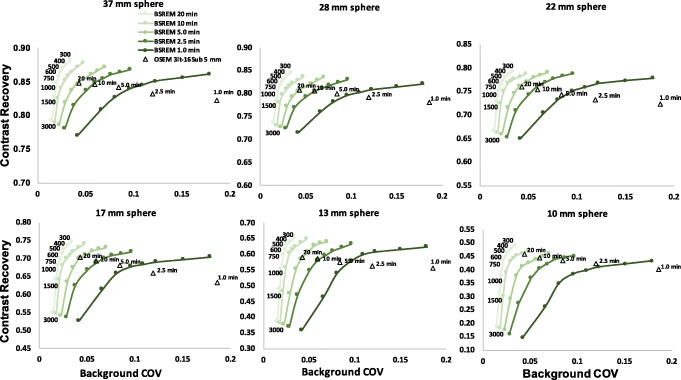
Contrast recovery of BSREM (***β*** = 300–3000, including TOF and PSF) and TOF-OSEM (3 iterations, 16 subsets, post-filter 5.0 mm and PSF) as a function of the background coefficient of variation for different acquisition times (20, 10, 5.0, 2.5, and 1.0 min) and contrast ratio of 8:1. Different plots are shown with decreasing sphere diameter

The results for CR versus background COV of the image quality phantom for each sphere size and contrast ratio of 8:1 are shown in Fig. 1. All plots show a similar trend: the contrast increases when reducing the *β*-factor and the COV decreases as *β* increases in value.

**Fig. 2:**
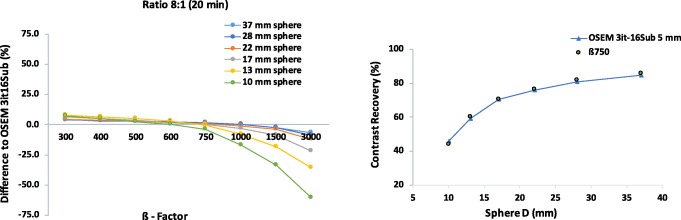
*Left*: difference relative to the contrast recovery of TOF-OSEM (3 iterations, 16 subsets, post-filter 5.0 mm and PSF) at high count statistics (20 min) for each sphere size as a function of the regularization parameters. *Right*: comparison of the contrast recovery as a function of the sphere size of BSREM with *β* = 750 (including TOF and PSF) and OSEM for 20 min acquisition. All plots correspond to a contrast ratio of 8:1

**Fig. 3:**
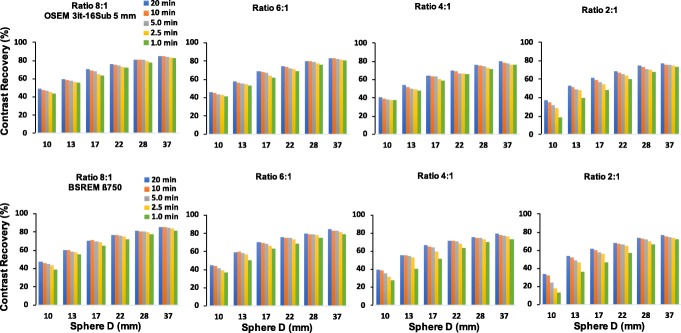
Comparison in terms of contrast recovery as a function of sphere size between (t*op*) TOF-OSEM (3 iterations, 16 subsets, post-filter 5.0 mm and PSF) and (b*ottom*) BSREM with *β* = 750 (including TOF and PSF) for different counting statistics and contrast ratios

**Fig. 4:**
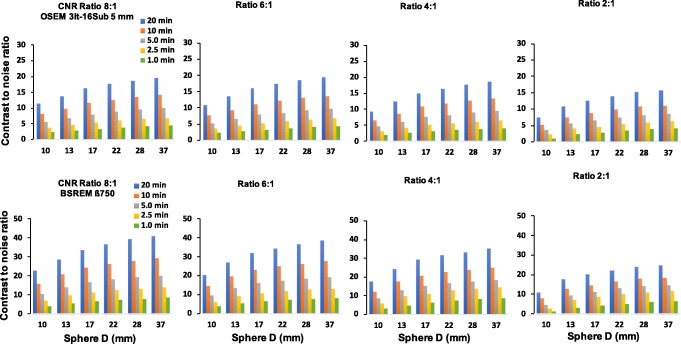
Comparison in terms of CNR as a function of sphere size between (top) TOF-OSEM (3 iterations, 16 subsets, post-filter 5.0 mm and PSF) and (Bottom) BSREM with *β* = 750 (including TOF and PSF) for different count statistics and contrast ratios. Note that the maximum scale value showed in the graphs are different between both reconstructions

Overall, the CR of BSREM reconstructions reaches a plateau with only a small gain when changing *β* from 500 to 300. This is especially the case for the phantom data with low count statistics. There is also a decrease in CR coefficients. Comparing CR of BSREM for different levels of regularization to the CR of OSEM reveals higher (or at least similar) contrast recovery for *β*-parameters down to 300, except for the smallest sphere. For the largest spheres (37, 28, 22, and 17 mm), the CR seems to reach a steady value, where its dependence on the *β*-parameter decreases. As the sphere size decreases, the convergence of the BSREM reconstructions appears to be dependent of the sphere size. The difference relative to OSEM for each sphere sizes as a function of the *β*-parameter is shown in Fig. 2. The CR of OSEM reconstruction under clinical settings at high count statistics (20 min) and contrast ratio of 8:1 match to the CR of BSREM with *β* = 750. Figure 3 shows a similar trend (as a function of the acquisition time) for the ratios 6:1 and 4:1. There is not a significant difference at high count statistics on the CR behavior between the ratios 8:1, 6:1, and 4:1, although the CR of BSREM decreases as the acquisition time reduces. This dependence on the count statistics seems to be more prominent at low count levels (1.0 min) and in the smallest sphere of the 2:1 ratio. The dependence on sphere size, counting statistics, and contrast ratio is also confirmed by the CNR analysis. Figure 4 presents the CNR of BSREM with *β* = 750 and OSEM reconstruction as a function of the sphere sizes for different count statistics and contrast ratios. For the ratios 8:1, 6:1, and 4:1, the CNR of *β* = 750 increased by a factor of 2 (for same count level) compared to OSEM reconstruction, although CNR decreases with the reduction of counting statistics and sphere size. The same trend was observed for the contrast ratio of 2:1, although there is a clear reduction in the CNR compared to the other ratios.

#### Noise properties

For reduced count statistics of the contrast 8:1 (reducing the acquisition time from 20 to 10, 5.0, 2.5, and 1.0 min), BSREM has in general lower COV than OSEM. Moreover, OSEM was more sensitive to noise compared to BSREM with large differences in COV between the different noise levels for the OSEM setting. When reducing the number of counts by a factor of 2, the COV can be controlled by increasing the *β*-parameter in the lower count dataset (losing contrast recovery). Similarly, the post-filter can be increased in OSEM. By comparing the reconstruction algorithms for different count levels, the following observations are made: The curves of BSREM at 50% of counts are always outperforming the curves of OSEM at the full 100% of counts. For the four largest spheres, the BSREM curves for 25% of the counts are also outperforming OSEM at 100% of counts. For any length of the study, the highest contrast recovery is observed for the smallest regularization parameter (300). The contrast recovery decreases with increasing *β*-parameter. In comparison with OSEM, the contrast is higher for *β* = 300–600 and comparable for *β* = 750 at 20, 10, and 5.0 min but slightly lower for 2.5 and 1.0 min acquisitions. Furthermore, taking as a reference the full dataset (20 min) reconstructed with OSEM (post-filter 5.0 mm), it is clear from Fig. 1 that the contrast (for all sphere sizes) with *β* = 300–600 is still higher for 20, 10, 5.0, and 2.5 min. For the smallest sphere, the optimal beta is around *β* = 300 and 400.

**Fig. 5:**
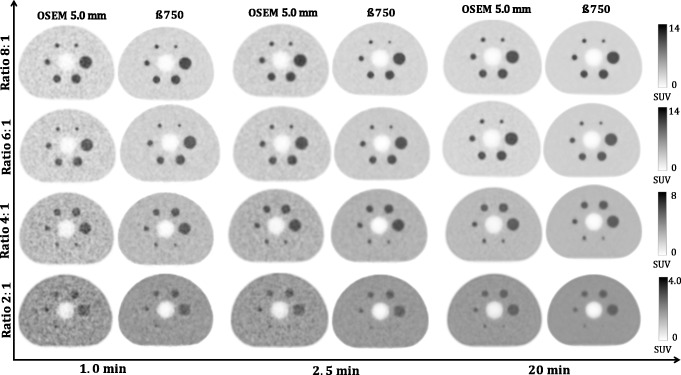
Qualitative evaluation of the transaxial images of the NEMA phantom of TOF-OSEM (3 iterations, 16 subsets, post-filter 5.0 mm and PSF) and BSREM with *β* = 750 (including TOF and PSF). Contrast ratios of 8:1, 6:1, 4:1, and 2:1 are shown in rows and acquisition times (1.0, 2.5, and 20 min) in column. Background level is 5.3 kBq/ml in all cases

The background COV comparison to OSEM is shown in Table 2. The COV of OSEM (20 min) is worse than the COV of BSREM with *β* = 750 for 20, 10 min, and 5 min. The COV of OSEM (10 min) is worse than the COV of BSREM with *β* = 750 for 10, 5.0, and 2.5 min. This represents a background COV reduction by a factor of 4. The quantitative noise reduction found in Table 2 is also visually confirmed by the reconstructions in Fig. 5. For all contrast ratios at 1.0, 2.5, and 20 min, BSREM (with *β* = 750) reconstructions appears to have better background COV compared to OSEM reconstructions. However, *β* = 750 has excessively smoothed the smallest sphere in the 2:1 ratio. This is also confirmed in Fig. 1, which suggests using a *β* value around 300 and 400 for small lesion at high count level. Increasing the *β*-factor leads to extra contrast loss and should only be done when the count level is low, and contrast can be traded in for reduced background COV.

**Table 2:** Comparison in terms of background COV between TOF-OSEM (3 iterations, 16 subsets, post-filter 5.0 mm and PSF) and BSREM with *β* = 750 for a volume of 26.52 ml at different count statistics. All values correspond to a contrast ratio of 8:1

	Background COV (26.52 ml)	
Time (min)	OSEM	β750
1.0	0.186	0.095
2.5	0.119	0.059
5.0	0.084	0.041
10	0.059	0.028
20	0.043	0.021

### Clinical data

**Fig. 6:**
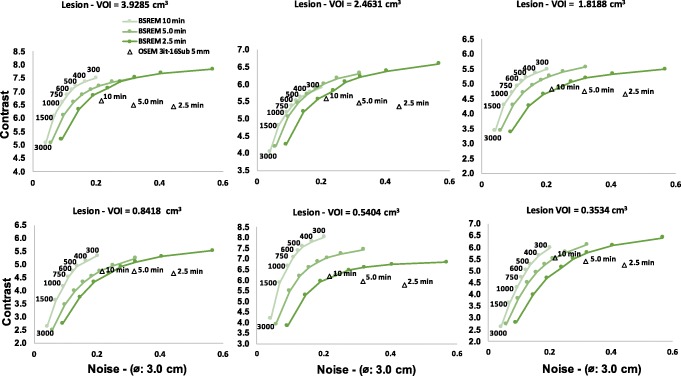
Contrast (SUV ratios) of BSREM (*β* = 300–3000, including TOF and PSF) and TOF-OSEM (3 iterations, 16 subsets, post-filter 5.0 mm and PSF) as a function of noise level of a large VOI (⌀, 3.0 cm) placed in normal liver for different acquisition times (10, 5.0, and 2.5 min). Different plots are shown with decreasing lesion volume

For the patient data, the contrast of the datasets with three different count levels (10, 5.0, and 2.5 min, representing each counting level a total of nine time ranges per bed position) was evaluated for six different lesion volumes as a function of noise level on the liver. The representative example of this patient data is presented in Fig. 6. The curves follow a similar trend as in the phantom reconstructions (Fig. 1). BSREM outperformed OSEM reconstructions in terms of noise levels with a lower noise level. Also, the largest difference is seen for bigger lesions where reduced noise is combined with higher contrast. The quantitatively measured values of the reference VOIs are shown in Table 3.

**Table 3:** Reference values measured in a healthy liver for OSEM and BSREM

Measure^b^	OSEM^a^				BSREM				
		β300	Β400	β500	Β600	β750	β1000	β1500	β3000
Volume (ml)	14.137								
SUV_mean_	2.212	2.187	2.184	2.192	2.193	2.197	2.193	2.204	2.212
SUV_SD_	0.480	0.441	0.362	0.301	0.272	0.241	0.206	0.152	0.092

^a^TOF PSF OSEM (3 iterations and 16 subsets and 5 mm Gaussian filter)

^b^Measured in a sphere placed in liver for 10 min (total) acquisition time

As shown in Table 4, the noise level measured in the liver with BSREM is clearly lower than for OSEM with post-filter. As excepted from the phantom data analysis, the noise in the liver of OSEM reconstruction (10 min) is worse than the noise of BSREM with *β* = 750 for 10 min and 5.0 min and is comparable to 2.5 min. Lower noise can be observed for BSREM in all other cases (also when lowering the counts with a factor 4). This leads to a noise reduction in the liver by a factor of 4. The SNR of BSREM with *β* = 750 and OSEM reconstruction are also shown in Table 4 for different count levels. A similar trend to phantom data presented in the Fig. 4 is observed SNR for BSREM with *β* = 750 (averaged for all lesion size) increased by a factor of 2 times for the same counting level. The SNR of both reconstructions decreases with reduction of counting statistics.

**Table 4:** Noise level of a large VOI (⌀, 3.0 cm) placed in the liver and the SNR with all lesion sizes averaged are presenting for TOF-OSEM (3 iterations, 16 subsets, post-filter 5.0 mm and PSF) and BSREM with *β* = 750 (including TOF and PSF). Reference values measured in a healthy liver

	Noise (14.137 ml)		SNR (lesion size averaged)	
Time (min)^a^	OSEM	β750	OSEM	β750
2.5	0.443	0.228	20.256	41.710
5.0	0.318	0.156	15.088	29.092
10	0.217	0.109	10.243	19.448

^a^Total of nine range of times per bed position

**Fig. 7:**
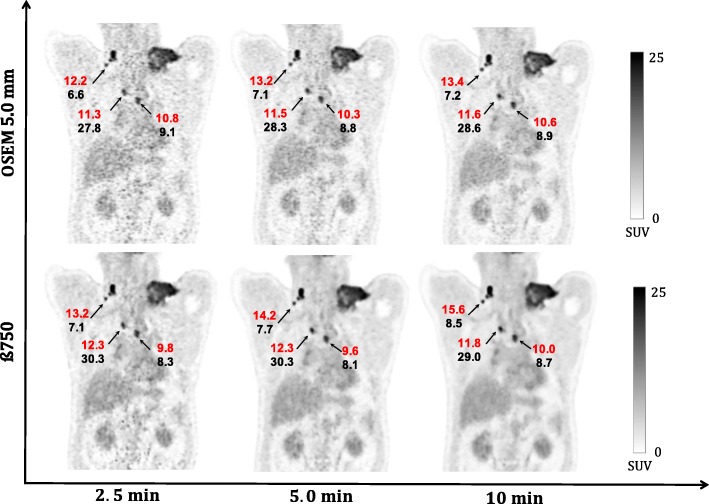
Coronal whole-body ^18^F-FDG PET images of patient with multiple lesions of different sizes of B cell lymphoma. The arrows indicate the SUV_mean_ (red) and MATVs (black) of the lesions. Images were reconstructed using BSREM with *β* = 750 (including PSF and TOF) and OSEM PSF TOF (3 iterations and 16 subsets and 5 mm gaussian filter) for 2.5, 5.0, and 10 min

Figure 7 shows quantitatively the SUV values of the evaluated whole-body ^18^F-FDG PET images of a patient with multiple lymphoma. In terms of SUV_mean_ and MATVs values for all lesion sizes, BSREM (with *β* = 750) reconstructions of 5.0 and 2.5 min are quantitatively similar to the OSEM reconstruction (10 min). Also, a qualitative visual evaluation has the same trends as the phantom reconstructions in Fig. 5.

Based on visual and quantitative differences, we found similar trends to the phantom data analysis (Figs. 1, 3, and 4). It is possible to reduce the count level of a clinical whole-body ^18^F-FDG PET/CT imaging with at least a factor of 2. Another similarity to consider, especially in terms of noise reduction without loss in contrast analyses, is that BSREM reconstruction increased the CNR and SNR by a factor of 2 (for both, phantom and the clinical data) compared to OSEM at same count level.

## Discussion

This study addressed the noise reduction performance of BSREM (Q.clear) compared to OSEM for both phantom and patient data acquired on the new Discovery MI 3-rings silicon photomultiplier-based TOF-PET/CT with sensitivity of 7.5 cps/kBq and axial FOV of 15 cm. Differences of the performance between OSEM and BSREM are expected due to the nature of the selective filtering of both algorithms, which can lead to a direct result on different anatomical information [16]. The aim was to investigate if the extent of noise can be reduced without a loss in image quality by using BSREM instead of OSEM.

The latest developments in detector technology and timing resolution of the digital PET/CT results in an increased sensitivity and count rate statistic compared with conventional PET/CT [18, 22]. These improvements combined with emerging developments in reconstruction methods lead to changes in the clinical routine which need to be considered and harmonized in order to obtain the most optimal image quality. This should be done in a shortest possible acquisition time, which is preferable in terms of patient care [23, 24].

BSREM reconstructions were compared to OSEM with PSF and TOF information using three iterations and 16 subsets with a FWHM of 5 mm, which were recommend by the manufacturer and are expected to be used in a general clinical setting. However, in most of the clinical centers, the reconstruction settings are chosen by the physicians based on the visual assessment of several PET scans.

Furthermore, the optimization of BSREM reconstructions has been previously investigated, such as Reynés-Llompart et al. [16] using a range of *β* 50–500 parameters on a BGO PET/CT. Other previous studies [14, 17, 24] have used a range of *β* 200–800 to examine the BSREM algorithm. In this work, we extended the range to *β* 300–3000 to assess in first, from a clinical point of view, which *β*-parameter has comparable contrast recovery to OSEM on a new DMI. Afterward, we evaluated qualitatively and quantitatively what is the impact on the noise under the condition of different contrast ratios and count statistics.

Regarding the contrast recovery and background COV for *equal count levels*, the phantom data analysis showed (Figs. 1 and 2) that for the four largest spheres (17, 22, 28, and 37 mm diameters), BSREM results in increased contrast recovery compared to OSEM. *β* = 750 (Fig. 2) has comparable resolution to OSEM reconstruction, but with a reduction of four times background COV (Table 2). Although *β* = 750 has a CR to OSEM, these results pointed out that the optimal penalization factor depends on the contrast ratio, acquisition time, and sphere size. This suggest that a high value of *β* can lead to a negative impact on the detectability of the small lesions. As presented in Fig. 1, the phantom results suggest an optimum *β* value between 300 and 400, which maximizes the CR and the CNR of the smallest sphere. This is also in agreement with the previous studies [14, 16, 23, 24].

BSREM outperforms OSEM with regards to the COV for all sphere sizes. The investigated clinical data showed a similar trend from the phantom study. For any length of the phantom data, the highest contrast recovery was found for a *β* = 300 (Figs. 1 and 6), but this value also has the highest background COV of any other BSREM reconstruction. These trends were also confirmed by the clinical data analysis (Fig. 6 and Table 4), where the noise level and contrast are higher for *β* = 300. The use of a lower FWHM value (FWHM < 5 mm) of the smoothing post-filter would have maximized the CR and the CNR of OSEM reconstruction; however, this would have increased the COV. In comparison with OSEM on the clinical data, BSREM reconstruction (especially for tumor lesions with VOI = 3.92 cm^3^ and VOI = 2.46 cm^3^) resulted in an increased tumor SUV_mean_, SUV_max_, and an improved contrast at a matched level of noise. The SNR of the average of all lesion sizes increased by a factor of 2 at the same count level (Fig. 4). However, for lesions smaller than 1.0 cm^3^ (Figs. 6 and 7), both reconstructions were equivalent in terms of SUV values. The same behavior was also found in the phantom data analyses (Figs. 1 and 2). A previous study [25] has reported that while PSF modeling commonly leads to visually enhanced images with higher contrast, it can simultaneously lead to notable edge artifacts affecting the quantification of small lesions. Thus, it is important to assess in which conditions is beneficial and warranted to use PSF modeling.

For *reduced count levels* based on phantom data, BSREM has in general a lower COV than OSEM. When reducing the number of counts by a factor of 2, the COV can be controlled by increasing the *β*-parameter in the lower count dataset (losing contrast recovery). A similar factor of 2 was observed in the clinical data. BSREM with a *β* = 750 increased SUV values and MATVs when compared to OSEM for the same acquisition time (Fig. 7). However, there was no difference in SUV values between OSEM (10 min) and BSREM (2.5 min), confirming that it is possible to have noise reduction with BSREM while preserving contrast.

Previous studies [23, 24, 26] have suggested *β* = 400 and *β* = 550 as an optimum factor. Another recent study with 45 patients in the initial stage of lung cancer has reported *β* values between 450 and 600 to be ideal for lung cancer [27]. Messerli et al. [28] highlight the importance for careful standardization of a *β* value when following-up non-small cell lung cancer. The optimum factor changes towards higher *β* values in patients who received a dose lower than 2 MBq/kg compared to patients who received doses higher than 2 MBq/kg. The higher *β* values appear to be more appropriate for patients with lower ^18^F-FDG doses.

In our study, the most favorable *β*-factor for both phantom and clinical data was in the same range with *β* = 750 at higher count levels. However, the choice of *β* might depend on several primary aspects, such as contrast, SNR, count statistics, radiation dose, or lesion detectability. Thus, the *β*-parameter should be chosen dependent on the requirements and context of the examination. Other aspects to consider are the acquisition duration and the axial FOV size of the TOF PET/CT used. According to EANM’s procedure, good clinical whole-body ^18^F-FDG images are usually obtained with an acquisition time of 3.0 min/bp [21]. The acquisition time reported evaluating the BSREM algorithm used three different count statistics varying from 3.0 to 1.0 min/bp reconstruction [22] which has somewhat a discrepancy compared to the range used 1.07, 0.34, and 0.17 min/bp in our study. This peculiar time ranges per bed position was chosen to evaluate the BSREM under the condition of reduced count statistics and, consequently, this decreased range would probably lead to an increase of the *β*-factor. The axial FOV of 20 cm of the PET/CT scanner would lead to improvements in sensitivity and count rate statistics compared to an axial FOV of 15 cm used in our study.

There are other limitations that should be considered in this study. All results from the clinical data analysis were taken based on a lymphoma patient with multiple lesions of different sizes. It is possible that an analysis of a larger and diversified group of patients would improve the results concerning the SUV lesion volume dependence. Consequently, it was not possible to evaluate the influence of the body mass index of the overweight patients on *β*-factor [26]. Additionally, there is a restriction concerning the ^18^F-FDG PET imaging tracer. The use of any other higher positron energy radioisotope would have led to a statistical uncertainty due to the random nature of radioactive emissions [29].

There is a minor risk of using BSREM when the primary requirement is to detect small lesions. Under the condition of higher count statistics, the combination of TOF-PSF-OSEM would lead to a comparable lesion detectability to BSREM, but if OSEM reconstruction is adopted, caution should also be taken regarding the increase of noise with the increase of the number of iterations. Lowering the counts by a factor of 2–4 (e.g., from 10 min to 2.5 min), BSREM would therefore lead to a comparable contrast recovery, CNR, background COV, and SUV values than TOF-PSF-OSEM reconstructions at higher count statistics. On the other hand, this reduction allows clinicians to reduce the PET activity needed for many exams, benefiting especially young patients. In general, BSREM outperforms OSEM reconstructions allowing noise reduction without losing data information.

## Conclusion

Penalized-likelihood BSREM reconstruction improves image quality and allows noise reduction by a factor of 2–4 while preserving contrast compared to OSEM reconstructions. Lowering of the injected dose or shortening the acquisition time is therefore possible by introducing regularization in the image reconstruction without a loss in image quality.

## Data Availability

The datasets used and/or analyzed during the current study are available from the corresponding author on reasonable request.

## Abbreviations

bp: Bed position
BSREM: Block sequential regularized expectation maximization
CR: Contrast recovery
CT: Computed tomography
FDG: Fluorodeoxyglucose
FOV: Field of view
GE: General Electric Company
LBS: Lutetium-based scintillator
MLEM: Maximum likelihood expectation maximization
NECR: Noise equivalent count rate
NEMA: National Electrical Manufacturers Association
OSEM: Ordered subset expectation maximization
PET: Positron emission tomography
PSF: Point spread function
ROI: Region of interest
SiPM: Silicon photomultipliers
SUV: Standardized uptake value
TOF: Time of flight
VOI: Volume of interest
